# Two-year randomized clinical evaluation of catechin doped in a universal adhesive on non-carious cervical lesions restorations

**DOI:** 10.1007/s00784-026-07282-z

**Published:** 2026-11-14

**Authors:** Nadine Luísa Guimarães Albuquerque, Cecília Atem Gonçalves de Araújo Costa, Ana Laura Mendes Mota, Vicente de Paulo Aragão Saboia, Juliano Sartori Mendonça, Sérgio Lima Santiago

**Affiliations:** 1 Graduate Program in Dentistry, Faculty of Pharmacy, Dentistry and Nursing, Federal University of Ceará, Rua Monsenhor Furtado s/n, Fortaleza, Ceará 60430-655 Brazil; 2 Department of Restorative Dentistry, Faculty of Pharmacy, Dentistry and Nursing, Federal University of Ceará, Rua Monsenhor Furtado s/n, Fortaleza, Ceará 60430-655 Brazil

**Keywords:** Catechin, Dentin bonding, Matrix metalloproteinases, Non-carious cervical lesions, Universal adhesive

## Abstract

**Objectives:**

This randomized, blinded clinical trial evaluated the 24-month clinical performance of a universal adhesive (Single Bond Universal) containing 0.1 wt% epigallocatechin-3-gallate (EGCG) for the restoration of non-carious cervical lesions (NCCLs using self-etch and etch-and-rinse strategies.

**Materials and methods:**

A total of 160 restorations were placed in 29 patients and randomly allocated to four groups: etch-and-rinse (ER), etch-and-rinse with EGCG (ER-EGCG), self-etch (SE), and self-etch with EGCG (SE-EGCG). EGCG was directly incorporated into the adhesive, which was applied according to the manufacturer’s instructions. All lesions were restored with a nanofilled composite resin (Filtek Z350XT) and evaluated at baseline and after 6, 12, 18, and 24 months according to the World Dental Federation criteria. Data were analyzed using the Kruskal–Wallis and McNemar tests (α = 0.05).

**Results:**

Participant recall rates were 100% at baseline and 6 months, and 93.1%, 89.7%, and 72.4% at 12, 18, and 24 months, respectively. No significant differences were observed among groups for any evaluated clinical criterion throughout the 24-month follow-up (*p* > 0.05). At 24 months, retention rates were 90% for SE and SE-EGCG and 100% for ER and ER-EGCG. No significant differences were observed between baseline and 24-month evaluations within any group (*p* > 0.05). Incorporation of 0.1 wt% EGCG into the universal adhesive did not compromise the clinical performance of NCCL restorations over 24 months, regardless of the bonding strategy.

**Conclusions:**

Incorporation of 0.1 wt% EGCG into a universal adhesive did not affect the clinical performance of NCCL restorations over 24 months. Clinical Relevance: Incorporation of 0.1 wt% EGCG into a universal adhesive did not improve the clinical performance of NCCL restorations over 24 months compared with the unmodified adhesive. However, EGCG incorporation did not adversely affect restoration performance when the adhesive was used with either etch-and-rinse or self-etch strategies.

## Introduction

Marginal deterioration and secondary caries remain the leading causes of composite resin restoration replacement [1, 2] and are closely associated with progressive degradation of the resin–dentin interface [3]. This degradation is a multifactorial process initiated by incomplete infiltration of adhesive monomers into the demineralized collagen network [4–6], leaving exposed collagen fibrils susceptible to both hydrolytic degradation, promoted by water sorption, and enzymatic degradation [7]. Endogenous matrix metalloproteinases (MMPs) [8, 9] and cysteine cathepsins play a central role in this process, promoting progressive degradation of the collagen matrix and compromising hybrid layer integrity [10, 11].

In addition to biological degradation, recent evidence has demonstrated that adhesive interface instability also results from hydrolytic degradation of adhesive polymers, water sorption, and mechanical fatigue, which act simultaneously during clinical aging and reduce the long-term durability of the resin–dentin bond [3, 4, 12]. Despite the significant advances achieved with contemporary universal adhesives, these degradation mechanisms continue to compromise the long-term stability of adhesive restorations [13]. Therefore, several strategies have been proposed to enhance dentin bond strength, preserve hybrid layer integrity, and promote a more stable and durable resin–dentin interface [14, 15].

One strategy to improve the durability of the resin–dentin interface is to inhibit endogenous proteolytic enzymes, particularly matrix metalloproteinases (MMPs) and cysteine cathepsins, which contribute synergistically to collagen degradation within the hybrid layer [8, 16, 17]. Chlorhexidine digluconate has been extensively investigated for this purpose [18, 19]. However, concerns regarding its effects on host-cell viability and its limited substantivity to mineralized dentin have encouraged the investigation of alternative approaches [20–22]. Furthermore, clinical studies have not demonstrated improved restoration performance following the incorporation of 2% chlorhexidine digluconate into self-etch adhesives or dentin pretreatment, with follow-up periods of 24 and 36 months, respectively [23, 24].

Biomimetic strategies have consequently gained attention as potential approaches to preserve the dentin matrix while maintaining the functional properties of adhesive systems [25–31]. Among these approaches, plant-derived polyphenols are of particular interest because their chemical structures enable interactions with collagen through hydrogen bonding, hydrophobic interactions, and other non-covalent mechanisms [14, 26, 32]. These interactions may modify the mechanical and physicochemical properties of dentin and increase its resistance to degradation.

Grape seed extract, which is rich in proanthocyanidins, has been widely investigated as a natural dentin biomodifier. Previous studies have reported improvements in dentin mechanical properties and resistance to collagen degradation, suggesting a potential role in preserving the resin–dentin interface [14, 32]. However, the effects of polyphenols are not uniform and depend on factors such as molecular structure, concentration, solvent composition, pH, and application time [32–34]. Variations in molecular size, flexibility, and the distribution of hydroxyl and aromatic groups may influence interactions with collagen and, consequently, the extent of biomodification [35]. Therefore, the effects observed with one polyphenol cannot necessarily be extrapolated to other compounds within this chemical class [32].

In this context, epigallocatechin-3-gallate (EGCG), the major polyphenol found in green tea, has emerged as a promising bioactive molecule capable of inhibiting collagenolytic enzymes while simultaneously stabilizing collagen and preserving the physicochemical integrity of contemporary universal adhesive systems [36, 37]. The galloyl groups present in EGCG promote collagen cross-linking and limit collagenase access to active binding sites within collagen fibrils [38, 39]. These complementary mechanisms enhance the stability of the resin–dentin interface and support the development of collagen cross-linking strategies for adhesive dentistry. Recent studies have further demonstrated that EGCG can improve the physicochemical stability of adhesive systems without adversely affecting their degree of conversion, polymerization, or immediate bonding performance [25]. The application of EGCG as a dentin pretreatment for self-etch adhesive systems [40, 41], or etch-and-rinse systems [15], as well as its incorporation into primers and adhesive systems [42–44], has preserved immediate bond strength while improving the long-term stability of the resin–dentin bond. Moreover, EGCG has demonstrated significant collagen cross-linking potential by enhancing the mechanical properties of demineralized dentin [11, 44, 45].

A randomized clinical trial evaluating EGCG as a dentin pretreatment demonstrated that the application of this biomolecule did not impair the clinical performance of adhesive restorations [46]. However, this approach requires an additional clinical step, potentially increasing technique sensitivity and reducing its clinical applicability. Therefore, incorporating EGCG directly into adhesive systems appears to be a more clinically feasible strategy, combining biomodification with simplified adhesive procedures. Although EGCG-containing adhesive systems have been extensively investigated [43, 44, 47], these studies have been limited to in vitro settings. To the best of our knowledge, no randomized clinical trial has evaluated the clinical performance of EGCG-containing adhesive systems in composite restorations placed in non-carious cervical lesions (NCCLs).

Because etch-and-rinse and self-etch strategies produce different degrees of dentin demineralization and collagen exposure, the extent of collagen availability for EGCG-mediated cross-linking, and consequently its inhibitory effect on collagenolytic enzymes, may vary according to the bonding strategy employed [48]. Therefore, evaluating EGCG-containing adhesives under both approaches is essential to determine whether its clinical benefit is dependent on, or independent of, the adhesive strategy used.

Therefore, this randomized, blinded clinical trial aimed to compare the retention rates and clinical performance of EGCG-free and EGCG-containing universal adhesive systems applied using two bonding strategies (self-etch and etch-and-rinse) in the restoration of non-carious cervical lesions over a two-year follow-up period according to the World Dental Federation (FDI) criteria. The null hypothesis tested was that the incorporation of EGCG into universal adhesive systems, regardless of the bonding strategy employed, would not influence restoration retention or clinical performance after two years of clinical service.

## Materials and methods

### Experimental design

This clinical trial followed the CONSORT (Consolidated Standards of Reporting Trials) statement [3​9]. The study was conducted in accordance with the ethical principles of the Declaration of Helsinki and was approved by the Ethics Committee on Investigations Involving Human Subjects of the local university (approval code 1.784.381/2016). The trial was registered with the Brazilian Registry of Clinical Trials (ReBEC, RBR-78s6nh).

This study was randomized and blinded for the patients and two evaluators with an equal allocation rate among the procedures under evaluation. Patients who participated in the study signed an informed consent form and were submitted to non-carious cervical lesion restorations with a universal adhesive system and nanofilled resin. In the control groups, the adhesive system was applied in two strategies, as suggested by the manufacturer (i.e., self-etch or etch-and-rinse). The experimental adhesive formulations were prepared by directly incorporating 0.1% (w/w) EGCG into Single Bond Universal (3 M Oral Care, St. Paul, MN, USA) through manual blending, without the addition of an external solvent. The modified adhesive was applied using the same etch-and-rinse and self-etch protocols as the corresponding control groups. Previous investigations of catechin-containing adhesive formulations and the physicochemical characterization of the EGCG-modified universal adhesive provided the experimental rationale for this approach [25]. The composition, manufacturers, and application mode of the materials used in this study are presented in Table 1. The study was carried out in the clinic of the local university, Dentistry School, and the restorations were performed between January and March 2017.

**Table 1:** Manufacturers, composition, lot number and application technique of materials used in this study

Material	Manufacturer	Composition	Lot number	Application technique
Epigallocatechin- 3-gallate	Sigma- Aldrich, St Louis, MO, USA	≥ 80% (high-performance liquid chromatografy), from green tea	#SLBJ 5907 V	0.1% incorporated in adhesive
Filtek Z350 XT	**3MOral** Care, St. Paul, MN, USA	Treated silanized ceramics;	#142,174 #147,907	Up to 3 increments of composite resin, individually photopolymerized.
		Silane treated silica; UDMA;		
		Bisphenol A polyethylene glycol		
		Diether dimethacrylate;		
		Bisgma; Ceramics of zirconia;		
		Polyethylene glycol;		
		Dimethacrylate; Triethylene		
		Glycol and dimethacrylate		
Single Bond Universal	3MOral Care, St. Paul, MN, USA	Ethanol; 2-hydroxyethyl	#588,645 #577,056	**Etch-and-rinse strategy**: commonly used phosphoric acid etching (about 35%) to the tooth structure (enamel and dentin) and allow to react for 15 s. Rinse thoroughly with water and dry with water-free and oil-free air or with cotton pellets; do not over dry. Adhesive application with the disposable applicator and rub it in for 20 s. If necessary, rewet the disposable applicator during the treatment. Subsequently direct a gentle stream of air over the liquid for about 5 s until it no longer moves and the solvent has evaporated completely. Light cure for 10 s. **Self-etch strategy**: Adhesive application with the disposable applicator and rub it in for 20 s. If necessary, rewet the disposable applicator during the treatment. Subsequently direct a gentle stream of air over the liquid for about 5 s until it no longer moves and the solvent has evaporated completely. Light cure for 10 s.
		Methacrylate; bisgma;		
		Decamethylene dimethacrylate;		
		Silane treated silica; Water;		
		Acrylic; copolymer and itaconic		
		Acid; Canforquinone; 1,10-		
		Decanediol phosphate		
		Methacrylate; and N, N-		
		Dimethylbenzocaine.		

Abbreviations: *UDMA* Urethane dimethacrylate, *BisGMA* bisphenol A-glycidyl methacrylate

### Patient selection

Patients were included in this clinical trial based on the following criteria: (1) at least 18 years of age; (2) good general and oral health, with no periodontal disease; (3) presence of at least 20 teeth in occlusion; (4) need for at least four restorations of non-carious cervical lesions (erosion, abrasion, and/or abfraction); and (5) non-retentive lesions with no more than 50% of the margin located in enamel and the cervical margin in dentin. Subjects with poor oral hygiene and teeth with previous restorative procedures or interproximal caries were excluded.

**Fig. 1:**
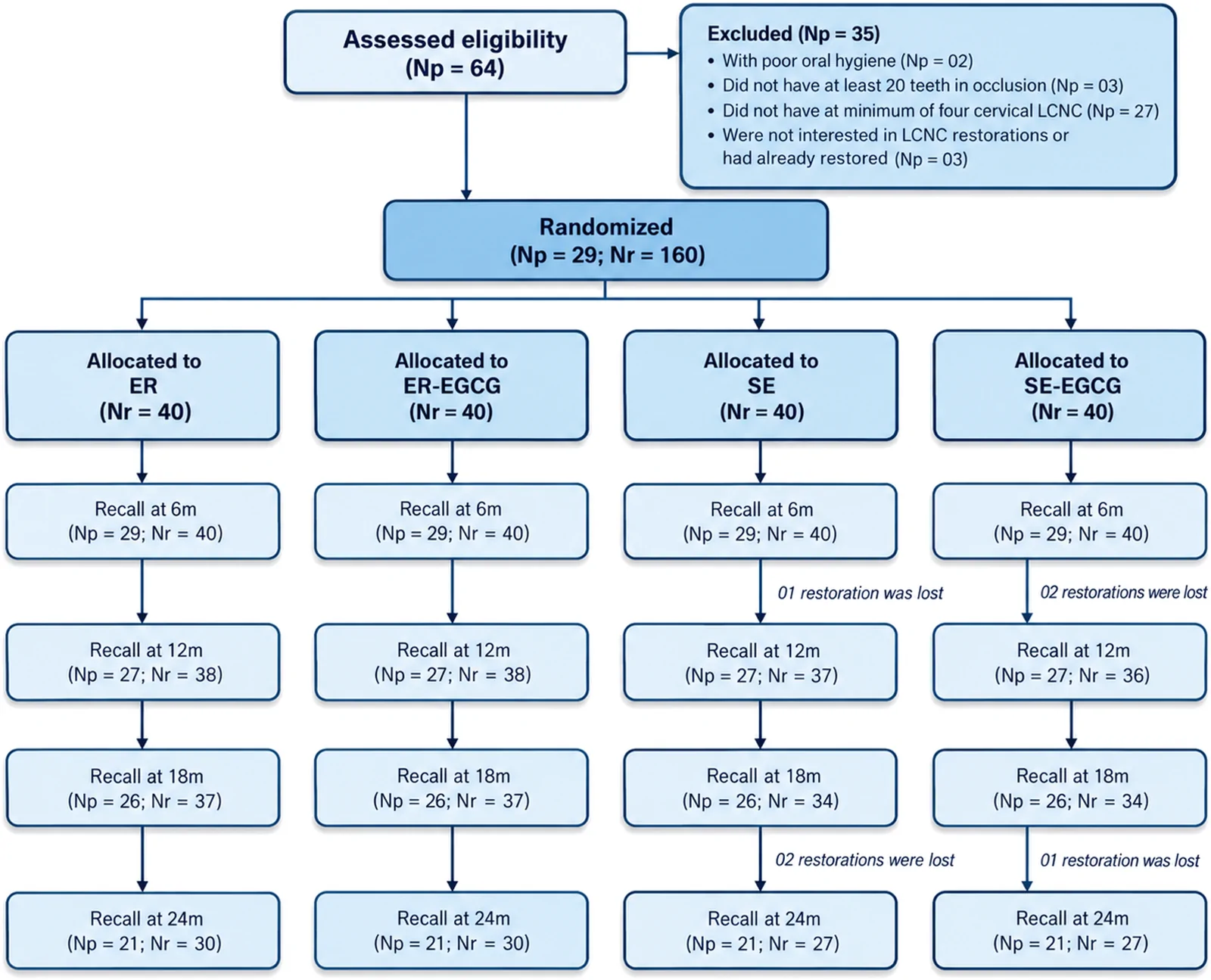
Flowchart diagram. Np, number of patients; Nr, number of restorations; ER, etch-and-rinse strategy; SE, self-etch strategy; EGCG 0.1%, epigalllocatechin-3-gallate added to the adhesive

Two calibrated dentists examined a total of 64 volunteers to determine eligibility. Based on these criteria, twenty-nine participants were selected for this study (Fig. 1).

### Sample size calculation and sample randomization

The sample size calculation was based on the retention rate of Single Bond Universal. Retention was reported to be 96% at the 18-month clinical follow-up [49]. Using a significance level of 5%, a power of 80%, and a two-sided test, the minimal sample size was 33 restorations in each group to determine a 25% difference among the tested groups. A 20% increase in the sample size was set, considering possible loss during the study.

A total of 160 non-carious cervical lesions from 29 participants were included in the study. Eighteen participants received four restorations, whereas eleven received eight restorations. Individual lesions were randomly allocated to the four experimental groups using a computer-generated randomization sequence prepared by a research team member who was not involved in the restorative procedures. Allocation concealment was maintained using sealed envelopes opened on the day of treatment. All restorations were performed by two calibrated operators following standardized restorative protocols. Clinical evaluations were conducted by two independent, calibrated examiners who were not involved in treatment delivery.

### Restorative procedures

Before conducting any therapeutic procedures, a complete anamnesis was performed to check the patient’s general and oral health. Table 2 describes the distribution of the restorations, in terms of location and type of tooth, dimensions, degree of dentin sclerosis, presence of an antagonist, and preoperative sensitivity. Each patient received 4 or 8 restorations for a total of 160 restorations performed in one appointment by two calibrated operators. No cavity preparation was carried out, enamel margins were not beveled, and no mechanical retention was placed, following the American Dental Association (ADA) guidelines [50].

**Table 2:** Characteristics of non-carious cervical lesions (shape, cervicoincisal size of the lesion, degree of sclerotic dentin, presence of antagonist, presence of incisal/occlusal wear facets, preoperative sensitivity (air dry and spontaneous), and tooth and arch distribution

Characteristics of NCCL	Number of lesions			
	ER	ER-EGCG	SE	SE-EGCG
Shape (degree of angle)				
< 45	1	0	0	1
45–90	15	15	18	14
90–135	10	9	11	14
> 135	14	16	11	11
Cervico-incisal height (mm)				
< 1.5	5	3	6	0
1.5–2.5	15	26	16	20
2.5-4.0	19	11	17	19
> 4.0	1	0	1	1
Degree of sclerotic dentin				
1	34	32	30	31
2	5	5	8	8
3	1	3	2	1
4	0	0	0	0
Presence of incisal/occlusal wear facets				
Yes	9	16	13	16
No	41	24	27	24
Preoperative sensitivity (air dry)				
Yes	28	30	26	32
No	12	10	14	8
Preoperative sensitivity (spontaneous)				
Yes	5	6	8	5
No	35	34	32	35
Tooth Distribution				
Incisors	6	7	9	4
Canines	4	11	4	5
Premolar	30	18	25	26
Molar	0	4	2	5
Arch distribution				
Maxillary	18	22	20	22
Mandibular	22	18	20	18

Initially, the operators determined the proper shade using a shade-selection guide (VitaPan Classic, Vita Zahnfabrik, Bäd Sackingen, Germany) and applied local anesthesia of the teeth with a 3% mepivacaine solution (Mepisv, Nova DFL, Rio de Janeiro, RJ, Brazil). The lesions were cleaned with pumice and water (SSWhite, Petrópolis, RJ, Brazil), followed by rinsing and drying.

Cotton-rolls isolation was carried out with a labial retractor (Arc Flex-FGM, Joinvile, SC, Brazil) and a retraction cord #00 (Ultrapack, Ultradent, Indaiatuba, SP, Brazil). Then, the NCCLs received the Single Bond Universal adhesive system (3 M Oral Care) applied in different modes: an ER and SE (etch-and-rinse and self-etch, respectively), suggested by the manufacturer and in experimental groups (ER-EGCG and SE-EGCG), following the same two protocols but with 0.1% EGCG w/w incorporated in adhesive. After adhesive application, Filtek Z350XT composite resin (3 M Oral Care) was placed in up to three increments, each being photopolymerized for 20 s with a 500mW/cm^2^ light-curing unit (Vip Junior, BISCO Inc., Schaumburg, IL, USA). The retraction cord was removed, and the final contouring and finishing of the restorations were immediately performed. The initial finishing was conducted with F and FF diamond tips (KG Sorensen, São Paulo, SP, Brazil), and 12- and 30-blade Multilayer Carbides burs (Microdont, São Paulo, SP, Brazil). The polishing was done with Sof-Lex Pop-On abrasive discs (3 M Oral Care).

High-resolution photographs were taken in two positions (vestibular and mesial) before the restoration at baseline and each recall using a digital camera (Canon, EOS, 60D, Canon Macro EF 100 mm lens).

### Clinical evaluation and statistical analysis

Two independent and calibrated evaluators not involved with the restoration procedures assessed the restorations immediately after completing the finishing and polishing procedures (baseline) and after 6, 12, 18 and 24 months of clinical service using the FDI criteria (World Dental Federation) [51, 52]. For each property, only the most relevant items were selected to evaluate the restorations’ clinical performance [50]. Restoration performance was assessed according to the FDI clinical criteria using a five-point ordinal scale. Scores 1, 2, and 3 were considered clinically acceptable, whereas scores 4 and 5 were classified as clinically unacceptable. Loss of restoration retention was recorded as a failure event. Cumulative failure percentages were calculated as [(PF + NF)/(PF + RR)] × 100, where PF represents previously recorded failures, NF represents new failures identified at the current recall, and RR represents restorations examined at that recall. Clinical scores were compared among the four experimental groups at each evaluation period using the Kruskal–Wallis test. Within-group comparisons between baseline and subsequent evaluations were performed using the McNemar test. Interexaminer agreement was assessed using Cohen’s kappa coefficient. Statistical significance was established at 5%.”

## Results

Thirty-five of the 64 patients were not enrolled in the study because they did not fulfill the inclusion criteria. Thus, 29 subjects (aged 28 to 55 years; 14 females and 15 males) were selected. Among them, 18 patients received four restorations, and 11 patients received eight restorations. All characteristics of the restored lesions are displayed in Table 2.

All participants completed the baseline and six-month assessments. Participant recall rates were 93.1%, 89.7%, and 72.4% at 12, 18, and 24 months, respectively. Participants who did not attend subsequent recalls could not be located. Restoration failures documented during clinical evaluations were distinguished from restorations for which follow-up observations were unavailable. Cohen’s Kappa statistics showed excellent inter-examiner agreement at the 6- and 18-month recalls (K = 0.91) and at the 12- and 24-month recalls (K = 0.90).

Data for all evaluated criteria at baseline and at all follow-up periods are summarized in Table 3. Pooled scores 1 + 2 and score 3 represent restorations classified as clinically acceptable, whereas scores 4 and 5 represent restorations considered clinically unsatisfactory.

**Table 3:** Number of evaluated restorations for each group (ER [etch-and-rinse, control]; SE [self-etch, control]; ER-EGCG [etch-and-rinse, 0.1%EGCG]; SE-EGCG [self-etch, 0.1%EGCG]) classified according to the FDI criteria at baseline, 6, 12, 18 and 24 months

Criteria	Group	Baseline				6-Mo				12-Mo				18-Mo				24-Mo			
		Scores				Scores				Scores				Scores				Scores			
		1 + 2	3	4	5	1 + 2	3	4	5	1 + 2	3	4	5	1 + 2	3	4	5	1 + 2	3	4	5
Marginal Discoloration	**ER**	40	0	0	0	40	0	0	0	38	0	0	0	37	0	0	0	30	0	0	0
	**SE**	40	0	0	0	38	1	0	0	36	1	0	0	33	1	0	0	26	1	0	0
	**ER-EGCG**	40	0	0	0	40	0	0	0	38	0	0	0	37	0	0	0	30	0	0	0
	**SE-EGCG**	40	0	0	0	38	0	0	0	36	0	0	0	34	0	0	0	27	0	0	0
Kruskal Wallis			*p* = 1.000				*p* = 0.5749				*p* = 0.3875				*p* = 0.3652				*p* = 0.3586		
Retention/ Failure	**ER**	40	0	0	0	40	0	0	0	37	0	1	0	35	1	1	0	29	1	0	0
	**SE**	40	0	0	0	38	1	0	1	35	2	0	1	31	3	0	3	25	2	0	3
	**ER-EGCG**	40	0	0	0	40	0	0	0	38	0	0	0	37	0	0	0	30	0	0	0
	**SE-EGCG**	40	0	0	0	38	0	0	2	36	0	0	2	34	0	0	3	27	0	0	3
Kruskal Wallis			*p* = 1.000				*p* = 0.2533				*p* = 0.3324				*p* = 0.0640				*p* = 0.0697		
Marginal Adaptation	**ER**	40	0	0	0	40	0	0	0	38	0	0	0	35	1	1	0	29	1	0	0
	**SE**	40	0	0	0	39	0	0	0	35	2	0	0	33	1	0	0	27	0	0	0
	**ER-EGCG**	40	0	0	0	40	0	0	0	38	0	0	0	37	0	0	0	30	0	0	0
	**SE-EGCG**	40	0	0	0	38	0	0	0	36	0	0	0	34	0	0	0	27	0	0	0
Kruskal Wallis			*p* = 1.000				*p* = 1.000				*p* = 0.1071				*p* = 0.3110				*p* = 0.4235		
Caries	**ER**	40	0	0	0	40	0	0	0	38	0	0	0	37	0	0	0	30	0	0	0
	**SE**	40	0	0	0	39	0	0	0	37	0	0	0	34	0	0	0	27	0	0	0
	**ER-EGCG**	40	0	0	0	40	0	0	0	38	0	0	0	37	0	0	0	30	0	0	0
	**SE-EGCG**	40	0	0	0	38	0	0	0	36	0	0	0	34	0	0	0	27	0	0	0
Kruskal Wallis			*p* = 1.000				*p* = 1.000				*p* = 1.000				*p* = 1.000				*p* = 1.000		
Postoperative sensitivity	**ER**	39	1	0	0	40	0	0	0	38	0	0	0	37	0	0	0	30	0	0	0
	**SE**	39	1	0	0	38	1	0	0	37	0	0	0	34	0	0	0	27	0	0	0
	**ER-EGCG**	40	0	0	0	40	0	0	0	38	0	0	0	37	0	0	0	30	0	0	0
	**SE-EGCG**	40	0	0	0	38	0	0	0	36	0	0	0	34	0	0	0	27	0	0	0
Kruskal Wallis			*p* = 0.5698				*p* = 0.3877				*p* = 1.000				*p* = 1.000				*p* = 1.000		

**Fig. 2:**
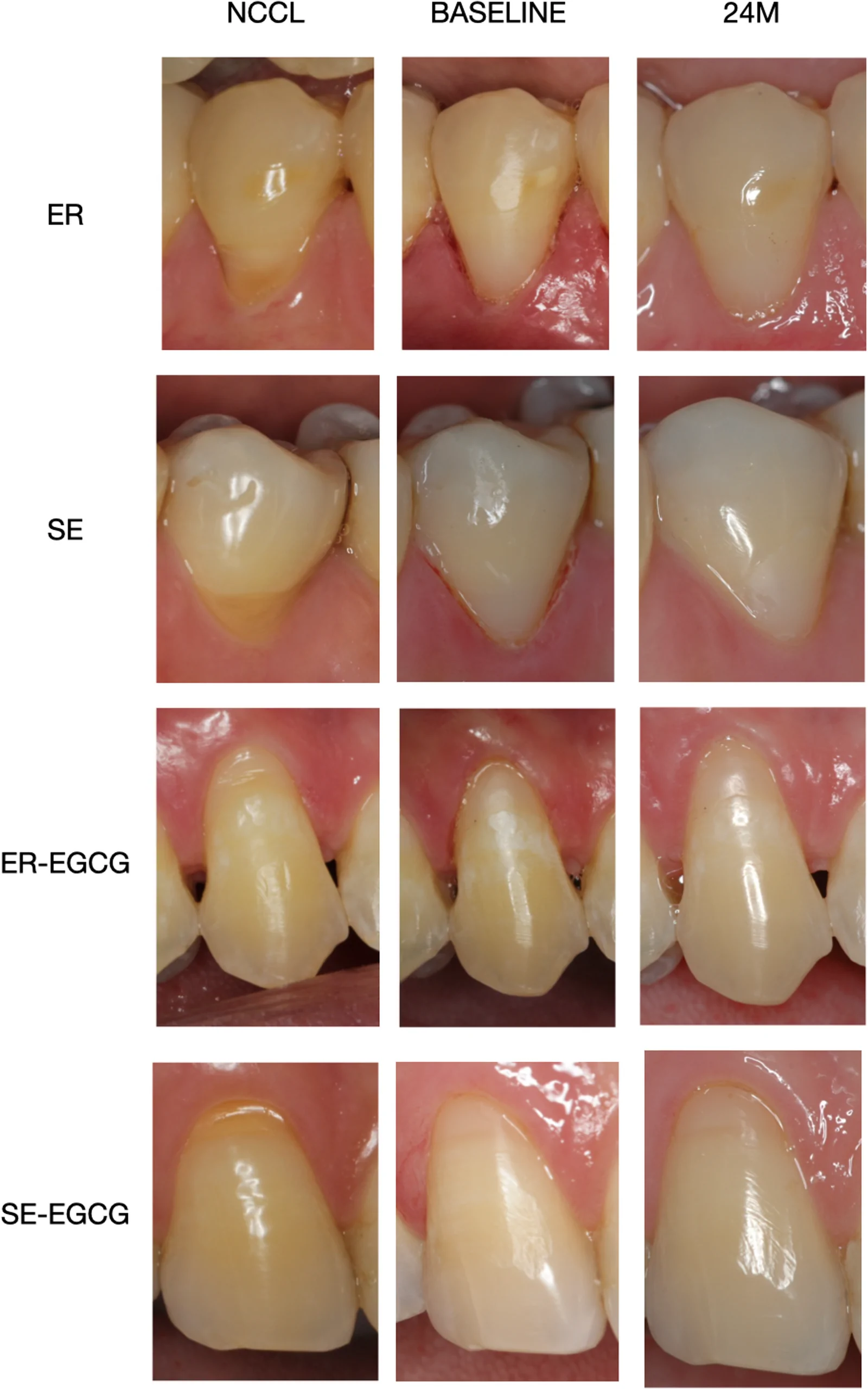
Noncarious cervial lesions from each group and clinically satisfactory restorations at baseline and 24 months

Three restorations were lost by the 6-month recall (one for SE and two for SE-EGCG), and three more were lost by the 18-month recall (two for SE and one for SE-EGCG). The 24-month retention rates were 90% for SE and SE-EGCG and 100% for ER and ER-EGCG, with no statistical difference among groups (*p* = 0.0697) or within each group when baseline and 24-month results were compared (*p* > 0.05; Table 3). At the 24-month recall, three restorations showed acceptable material chip fracture (one for ER and two for SE) without affecting marginal integrity (score 3). For SE, acceptable chip fracture (score 3) was observed in one restoration at the 6-month recall, two at the 12-month recall, and three at the 18-month recall. For ER, one restoration showed acceptable chip fracture at the 18-month recall, and another showed a bulk fracture with partial loss (score 4) at both the 12- and 18-month recalls. No restorations in the EGCG groups received scores of 3 or 4. Figure 2 presents representative clinical photographs of non-carious cervical lesion restorations performed using the etch-and-rinse (ER), etch-and-rinse with EGCG (ER-EGCG), self-etch (SE), and self-etch with EGCG (SE-EGCG) strategies at baseline and 24-month follow-up evaluations.

Regarding marginal adaptation, at the 12-month recall, two SE restorations showed gaps (< 150 μm) that could not be removed with an explorer (score 3). At the 18-month recall, two restorations scored 3 (one each for ER and SE), and one ER restoration was classified as clinically unsatisfactory due to dentin exposure (score 4). At 24 months, one restoration scored 3. No significant differences were detected between groups at the 6-, 12-, 18-, and 24-month recalls (*p* > 0.05).

Moderate marginal staining (score 3) was observed in only one SE restoration across all recall periods, with no significant differences among groups at 24 months or within each group when baseline data were compared with all recall periods (*p* > 0.05).

Two restorations had postoperative sensitivity (score 3) at baseline (one ER and one SE) and, one of these (SE) persisted at 6 months, with no statistical difference among groups (*p* > 0.05). No restoration showed postoperative sensitivity at 12, 18, or 24 months. Finally, no caries were observed during the 24 months of clinical follow-up.

## Discussion

Currently, to improve the quality of the adhesive interface, studies have emphasized the modification of adhesive systems through the incorporation of enzymatic inhibitors to protect the bond durability of dental adhesives [12, 41, 53]. Epigallocatechin-3-gallate (EGCG), a flavonoid derived from Camellia sinensis, is a natural collagen cross-linking agent capable of enhancing the mechanical properties of dentin and reducing host-derived MMP-mediated degradation [54]. More recently, EGCG has also been shown to inhibit recombinant MMP-2 and MMP-8, as well as matrix-bound endogenous proteases within the hybrid layer, an effect attributed to hydrogen-bond formation between its galloyl moiety and the catalytic domain of these enzymes [39].

In vitro studies have demonstrated that EGCG preserves adhesive bond strength when applied with an ER adhesive system [15, 55], an SE adhesive system [41, 53], or incorporated directly into the adhesive [35, 44, 3​6, 45]. Du et al. [41] showed that immediate bond strength increased after incorporation of 0.01–0.03% EGCG, improving adhesive durability. When applied as a dentin pretreatment, EGCG increased µTBS after 6 months [40] and preserved resin-dentin bond strength for up to 12 months [47]. It has also been shown to improve the mechanical properties of demineralized dentin, suggesting a collagen cross-linking effect [25, 44, 26, 4​5] and the addition of EGCG-methacrylate monomers to a self-etch adhesive reduced gelatinolytic activity within the hybrid layer without compromising bond strength [44]. Despite these promising laboratory findings, no clinical studies have evaluated the effect of EGCG-doped adhesives on the durability of resin-dentin bonds. Therefore, this study sought to evaluate the effect of EGCG incorporation into a universal adhesive on the retention rate of NCCL restorations.

The incorporation of catechin-derived compounds into adhesive formulations has been investigated as a strategy to improve the physicochemical properties and durability of the resin–dentin interface. Guimarães et al. [25] characterized the same 0.1 wt% EGCG-modified universal adhesive formulation investigated in the present clinical trial, reporting preserved degree of conversion and flexural strength, reduced water sorption, and improved bonding stability under selected experimental conditions. These findings provided a relevant preclinical rationale for evaluating the clinical performance of the modified adhesive.

Since no cavity preparation was performed, the restorations’ actual dentin-bonding capacity could be assessed. NCCLs are considered ideal lesions for testing the clinical effectiveness of adhesives [54, 56, 57], given their combination of enamel and dentin substrates, lack of macromechanical retention, and relatively low C-factor, which minimizes the influence of the composite resin’s mechanical properties on the outcome [56]. All restorations were placed under cotton-roll isolation to reflect routine clinical practice.

Regarding the mode of EGCG application — as a pretreatment solution or incorporated into the adhesive — previous in vitro studies [15, 40, 41, 47, 53, 55] have not established which approach is more effective. Incorporating EGCG directly into the adhesive may simplify the clinical protocol by reducing operative steps and, as previously described for chlorhexidine [12], allows the inhibitor to contact dentin simultaneously with the acidic monomers responsible for demineralization and infiltration, potentially reducing the moisture-control challenges associated with a separate pretreatment step. Breschi et al. [17] reported that several strategies have been developed to preserve the hybrid layer over time, including different approaches to inhibiting endogenous enzymatic activity.

In the current study, Single Bond Universal — an ultra-mild adhesive (pH ≈ 2.7) — was applied using both etch-and-rinse and self-etch strategies for the restoration of NCCLs [58]. No statistically significant differences in clinical performance were observed between strategies, consistent with previous in vitro studies [59, 60] and randomized clinical trials [46, 52, 61, 62]. Retention rates were numerically, though not significantly, lower in the SE and SE-EGCG groups (90%) than in the ER and ER-EGCG groups (100%) at 24 months. Because this pattern occurred both with and without EGCG, it appears more closely related to the bonding strategy itself than to EGCG incorporation. This is in line with a recent systematic review and meta-analysis of RCTs on NCCL restorations with universal adhesives, which reported a significantly higher risk of retention loss with the SE strategy than the ER strategy at 12 and 18/24 months, with no strategy-related differences in marginal discoloration or adaptation [63]. Overall, Single Bond Universal containing 0.1% EGCG showed similar clinical performance to the control groups at the 24-month recall, with no significant differences in retention or any other evaluated parameter, regardless of bonding strategy; therefore, the null hypothesis could not be rejected, as EGCG incorporation did not improve the clinical performance of composite resin restorations in NCCLs bonded with either strategy.

This strategy-related trend is also consistent with a four-year RCT using the same adhesive brand (Scotchbond Universal), in which the self-etch group showed a significant reduction in retention from baseline, whereas selective enamel etching maintained stable retention throughout follow-up [4], suggesting that strategy-related differences may persist — or even widen — beyond the 24-month period evaluated here. However, this tendency does not appear to be universal: a 7.5-year split-mouth RCT with Futurabond U found no significant retention differences among self-etch, selective-etch, and etch-and-rinse strategies even at the final follow-up [13]. The authors attributed this to the adhesive’s pH and chemical reactivity, proposing that sufficiently acidic universal adhesives can adequately demineralize the smear layer and underlying dentin independent of prior acid conditioning [13]. This consideration may be particularly relevant here, since Single Bond Universal is classified as an ultra-mild adhesive (pH ≈ 2.7) [58]; its lower intrinsic acidity may increase dependence on prior phosphoric-acid conditioning, offering a plausible explanation for the numerically higher retention observed with the etch-and-rinse strategy in the present study.

In the current study, EGCG (0.1% w/w) was incorporated directly into Single Bond Universal differing from previous protocols in which EGCG was first dissolved in ethanol before being added to the adhesive [41, 53]. Direct incorporation avoids additional manipulation of the formulation and potential changes in the solvent/monomer ratio. Notably, the same 0.1% EGCG-doped formulation used in this trial was previously evaluated in vitro by our research group [25], which EGCG did not adversely affect degree of conversion, solubility, or flexural strength, while reducing water sorption and increasing the modulus of elasticity; the SE-EGCG group also showed significantly higher µTBS than the SE control after 12 months of water storage, whereas ER-EGCG showed a progressive reduction in bond strength over the same period. Similarly, Sousa et al. [64] showed that experimental etch-and-rinse (E&R) and self-etch (SE) adhesives doped with 1% epigallocatechin-3-gallate (EGCG) adsorbed on hydroxyapatite (HAp) nanoparticles proved effective in dentin bonding, even after aging. Additionally, this concentration increased flexural strength in the E&R system without interfering with polymerization. These prior findings support the interpretation of the present clinical results: although EGCG did not significantly improve retention or other clinical parameters at 24 months, the SE-EGCG group maintained the same retention rate as the SE control (90%) rather than a lower one, consistent with the greater bond stability previously observed for this formulation in vitro — although whether this translates into a measurable clinical benefit over longer follow-up remains to be determined.

A complementary in vitro study using the same adhesive in self-etch mode found that 0.1% EGCG alone preserved µTBS after six months of water storage, with no significant difference from the unmodified control, whereas combining EGCG with a remineralizing agent (β-tricalcium phosphate) provided no additional benefit and was associated with increased nanoleakage, attributed to particle aggregation impairing resin infiltration [48]. This suggests that EGCG’s protective effect on bond stability is a specific, non-additive property rather than one enhanced by combination with other bioactive strategies. Similarly, The same research team also reported comparable clinical outcomes in a two-year evaluation of EGCG incorporated into this adhesive system [46], reinforcing that the absence of a measurable clinical benefit from EGCG at 24 months is a reproducible finding across independent cohorts using the same material.

Restorations were evaluated using FDI criteria, given their sensitivity for detecting small differences in the clinical outcomes of NCCL restorations [62]. As noted above, three restorations each in the SE and SE-EGCG groups were considered clinically unacceptable due to loss of retention (score 5) at 24 months, yielding a 90% retention rate for both groups, versus 100% for ER and ER-EGCG (*p* > 0.05). This pattern of numerically, but not significantly, lower SE retention is consistent with other NCCL trials using the same similar adhesive brand Souza et al. [65] reported a progressive, time-dependent decline in retention when proanthocyanidins were incorporated into a two-step etch-and-rinse adhesive, falling from 98% (control) to 73% and 71% at 24 months for 2% and 5% concentrations, respectively — an effect attributed to interference with free-radical polymerization, resulting in more unreacted monomers, increased interfacial porosity, and greater susceptibility to hydrolytic degradation and marginal staining. This deleterious, concentration-dependent effect contrasts with the neutral clinical behavior of EGCG observed here, underscoring that the clinical performance of collagen cross-linkers and enzymatic inhibitors added to adhesive systems cannot be extrapolated from one polyphenol class to another, even when their in vitro mechanisms of action appear similar.

Regarding marginal discoloration and adaptation, all restorations in the EGCG groups were scored as clinically very good or good (scores 1 or 2). One SE restoration showed moderate, though esthetically acceptable, marginal staining, and one ER restoration required repair at the 18-month recall for unsatisfactory marginal adaptation; however, most marginal defects were small and clinically acceptable, with no relevant impact on overall outcomes.

None of the restorations exhibited caries. Marginal discoloration and secondary caries can be influenced by water sorption [66], as polymer swelling reduces interchain frictional forces and degrades the mechanical properties of the adhesive. Neri et al. [42] found that EGCG incorporation reduced solubility without affecting water sorption, degree of conversion, or flexural strength in a methacrylate-based adhesive — consistent with the more recent characterization of EGCG-doped Single Bond Universal, which likewise showed no adverse effect on degree of conversion or flexural strength, together with a reduction (rather than an increase) in water sorption [25].

In summary, incorporating 0.1% EGCG into Single Bond Universal did not significantly alter the clinical performance of NCCL restorations at 24 months, regardless of bonding strategy. The absence of a detectable EGCG effect is likely related to the relatively short follow-up period: the protective effect of EGCG against MMP-2, MMP-8, and bacterial collagenase has been mechanistically confirmed [39], and its incorporation into Single Bond Universal improved bond-strength stability specifically at 12 months in the SE mode [25] — a benefit that a 24-month clinical follow-up, assessed primarily through a categorical retention outcome, may not be sensitive enough to capture. Four-year data for the same self-etch/selective-etching strategies with this adhesive [4] further suggest that clinically meaningful divergences between strategies — and potentially between biomodified and unmodified adhesives — may only emerge well beyond the two-year mark, supporting the need for extended follow-up of the present cohort. This time-dependent divergence does not appear universal across all universal adhesives, however: a 7.5-year trial with a mild-pH system showed stable, overlapping survival curves across all bonding strategies throughout follow-up [13]​. This contrast suggests that the trajectory of strategy-related performance over time is likely material-dependent, reinforcing the rationale for extended follow-up of the present ultra-mild, EGCG-modified adhesive specifically — since comparable ultra-mild systems, rather than universal adhesives in general, have shown this pattern of late-emerging divergence.

Recent investigations have further highlighted the importance of long-term clinical evaluation when assessing the performance of universal adhesives and bioactive modifications. Ñaupari-Villasante et al. [13] reported long-term clinical outcomes from a 7.5-year randomized trial, emphasizing the relevance of adhesive formulation and application strategy to restoration longevity. Furthermore, Santiago et al. [48] investigated the physicochemical properties and interfacial morphology of a universal adhesive modified with a collagen cross-linker and a remineralizing agent. Collectively, these findings reinforce the need to interpret the potential benefits of bioactive adhesive modifications in relation to both their physicochemical behavior and their demonstrated clinical performance.

The findings of this study should be considered in light of some methodological aspects. First, although the 24-month follow-up provided relevant information on the clinical performance of EGCG-incorporated adhesives, longer observation periods may provide additional insights into their long-term behavior and restoration longevity. Second, participant retention at the 24-month evaluation was 72.4%, which may have influenced the precision of the estimates at the final follow-up. Third, because some participants received more than one restoration, observations may not be completely independent; however, this reflects the clinical setting and should be considered when interpreting restoration-level findings. Finally, the results apply specifically to the adhesive formulation, EGCG concentration, and clinical protocols evaluated in this study and may not necessarily extend to other adhesive systems or concentrations. Further clinical studies with longer follow-up and larger samples may help confirm the long-term clinical performance of EGCG-incorporated adhesives.

## Conclusion

The clinical behavior of the universal adhesive in NCCL restorations at 24-months did not depend on bonding strategy, and epigallocatechin-3-gallate did not negatively affect the retention rate of the restorations.

## Data Availability

No datasets were generated or analysed during the current study.

## Declarations

### Clinical trial registration

Registry: Brazilian Clinical Trials Registry (ReBEC). Registration number: RBR-78s6nh. Registration URL: https://ensaiosclinicos.gov.br/rg/RBR-78s6nh.
